# Feasibility and Acceptability Study of Virtual Reality Support Groups for Transgender and Gender Expansive Youth

**DOI:** 10.1089/jmedxr.2024.0040

**Published:** 2025-03-28

**Authors:** Nicolas Meade, Allura Ralston, Veronica Weser, Kimberly Hieftje, Christy Olezeski, Asher Marks

**Affiliations:** ^1^ Department of Psychiatry, Yale School of Medicine, New Haven, Connecticut, USA.; ^2^ Department of Pediatrics, Yale School of Medicine, New Haven, Connecticut, USA.

**Keywords:** transgender youth, social support, virtual reality, gender joy

## Abstract

Transgender and gender expansive (TGE) youth are facing unprecedented levels of social and structural stigmatization and discrimination. Antitransgender legislation almost exclusively focuses on limiting affirming supports for TGE youth by utilizing stigmatizing rhetoric surrounding transgender identities based on myths and disinformation. In the face of increasing societal oppression, TGE individuals report higher rates of experienced discrimination, violence, and social rejection. Interventions that promote individual and community resilience are vital to supporting the wellbeing of TGE youth. Virtual support groups and therapies for TGE youth may be essential as in-person access to affirming spaces continues to be limited. In this article, we present feasibility and acceptability data of a social virtual reality (VR) tool to support TGE youth for whom in-person supports may not exist and for whom video-based telehealth platforms may trigger dysphoria. Using a mixed-methods approach, we collected data from 10 TGE youth participants on the feasibility and acceptability of an eight-week, VR social support group, prior to transitioning to a peer-to-peer model based on overwhelming participant feedback. We posit that gender affirming VR support groups and therapies may help increase critical resilience factors for TGE youth that are being restricted from gender affirming supports and call for further research to investigate the usefulness of VR and other immersive platforms to support gender affirmation and community connection.

## Introduction

In the United States, over 600 antitransgender bills were introduced in 2024, nearly all of which specifically aim to limit the human rights of and social supports for transgender and gender expansive (TGE) youth.^1^ TGE is an inclusive term that encompasses individuals who do not exclusively identify with the sex assigned to them at birth. Along with the increase in antitransgender laws and harmful rhetoric, individual reports of interpersonal violence toward, discrimination against, and rejection of TGE individuals have also increased.^2^ Despite making up an estimated 2–7% of the population of youth in the United States,^3,4^ TGE youth have been subject to increasing societal stressors resulting in distinct increases in negative mental health symptoms, including rises in already elevated rates of suicidal ideation and attempts, depressive symptoms, and social isolation.^5,6^

Considering these increasing stressors, it is imperative to develop effective, supportive, and accessible resources for transgender and gender-expansive (TGE) youth in the United States. It is essential to understand the impacts of these risk factors on their well-being, as well as to acknowledge the protective factors that are already present and available. As resources for TGE youth continue to be limited, digital interventions and supports can serve as accessible ways to build community and cultivate gender joy.

### TGE risk and protective factors

#### Gender minority stress and resilience

Current models of gender minority stress and resilience conceptualize the unique external and internal factors that TGE individuals experience, as well as the impact of those experiences on overall health and wellbeing.^7,8^ Unlike their cisgender peers, TGE individuals encounter increased distal stressors such as violence, sexual assault, harassment, and identity invalidation.^7^ Notably, TGE youth report higher levels of harassment and violence in schools.^6^ Systemic stigmatization and restricted access to gender-affirming health care further compound these internal and external stressors.

#### Social rejection

One of the most significant affective impacts of social rejection is the internalization of negative social feedback, resulting in shame. For TGE youth, this manifests cognitively as the internalization of transphobia, the belief that gender variance is innately wrong or bad.^7^ Internalized transphobia presents as cognitions related to prejudice toward the self and is associated with increased depressive symptoms.^9^ The affective experience of shame is related to feelings of worthlessness and a negative evaluation of oneself.^10^

TGE youth’s self-esteem is heavily impacted by social acceptance or rejection. Thus, creating affirming environments is crucial to protect them from harassment in various social contexts. Positive community interactions and shared activities help safeguard their well-being, but finding supportive spaces is becoming harder as resources continue to be restricted.^11^

#### Gender joy

Gender joy and gender euphoria have been defined in a variety of ways in the literature including “a form of trans resilience,” “distress relief and wellness promotion,” and “distinct enjoyment and satisfaction.”^12–14^ However, it is important to understand that the terms, which are often synonymous, came from the TGE community and are therefore best defined by community members. In a survey of individuals across the gender spectrum, gender joy was defined as “happiness, joy, contentment, and the feeling of being righted,” but the most poignant description came from a participant that emphasized that gender joy “is literally lifesaving…it is what keeps trans folks alive.”^15^

### Digital interventions and virtual reality

#### Current challenges and potential solutions

Videoconferencing platforms continue to be explored as cost-effective and accessible ways for LGBTQ youth participate in mental health therapy groups.^16^ However, seeing oneself on camera has the potential to activate gender dysphoria for some TGE youth.^17^ Virtual reality (VR) presents a unique solution for TGE youth with gender dysphoria to connect with community members in real time. In virtually created or augmented space, TGE youth have an opportunity to interact with their peers without geographic constraints while embodying a more congruent presentation through the use of digital avatars.

VR uses a combination of body tracking and head mounted displays to block out external sights and sounds to completely immerse the user in a carefully curated and customized artificial environment. VR also allows users to share these experiences and environments together with others from anywhere in the world. In addition, the technology offers users the opportunity for embodiment within an avatar that interacts with other participants and can be highly customized to the user’s specifications, including the ability to adjust the pitch of users’ voices. Robust evidence exists for clinical applications of VR exposure therapy for a variety of anxiety disorders, eating disorders, and body image disturbances.^18,19^

#### Embodiment

Embodiment is the sense that an observed object is, in fact, one’s body or a part of one’s body, resulting from the integration of simultaneous sensations such as vision and proprioception.^20^ Under the right conditions, even noncorporal objects can become embodied.^21^ In social VR spaces, a VR user’s self-presentation is grounded in a one-to-one relationship between their physical body movements and their avatar. In this manner, avatars substitute for the visible body in VR, and although users “know” that avatars are not really them, avatars become embodied via simultaneous multisensory integration processes.^22^ Embodying an avatar can lead to a powerful sense of presence in VR, known as embodied presence.^23^

In VR, the presentation of self may be a different social identity or even a reflection of one’s true self unencumbered by the limitations of the offline world. In a recent qualitative exploration of self-presentation in social VR platforms, researchers interviewed noncisgender individuals for whom social VR provided a powerful embodied way to explore their gender identity in a safe and realistic environment.^24^

#### Virtual reality as a supportive solution

In current sociopolitical environments that restrict access to gender affirming care, the access to gender joy is also being limited. VR therapy may allow TGE youth with gender dysphoria to see themselves and be seen by others in a more authentic and consistent manner through the opportunity to design their own avatars. The evolution of avatar design has enabled the creation of both realistic and hyperexpressive avatars that can be used across platforms. VR-mediated groups have the potential to support TGE youth in feeling gender joy as they can exist virtually as avatars consistent with their gender identities and connect with other TGE youth in safe and confidential virtual spaces. This may result in increased pride in themselves and community connection, factors that are strongly associated with positive health outcomes for TGE individuals.^7,8^

## Materials and Methods

In this pilot study, we utilized a primarily qualitative approach to understand the acceptability, accessibility, and feasibility of VR support groups for TGE youth, as well as the VR technology itself (including the hardware and software). Prior to initiation of recruitment, the study was approved by the Yale School of Medicine’s Institutional Review Board.

### Participants

Participants who self-identified as transgender youth between the ages of 12 and 18 were recruited both online through an email sent to patient and caregiver listservs through the Yale Pediatric Gender Program and during in-person clinic visits. To participate in the study, participants needed to be English speaking and have consistent access to the internet. After a brief meeting with the young person and their caregiver to obtain informed consent, the first author provided the youth with an Oculus Quest 2 headset and engaged the participant in a brief orientation to the technology and VR platform. During the orientation, the participants were guided through the login processes for the headsets and social VR application. Participants were able to use the VR headsets in any location with adequate Wi-Fi signal and privacy. In total, 10 TGE youth between the ages of 13–18 were assigned to one of two pilot groups based on rolling admission. A total of 16 sessions were held, with each group attending eight sessions a piece. Group one was conducted between September 2022 and November 2022 and group two between March 2023 and May 2023. See Table 1 for demographic information.

**Table 1.** Self-Reported Participant Demographics and Characteristics

Characteristics	Mean or *n*
Age	15.9
Gender Identity	
Transmasculine agender	3
Nonbinary woman	2
Trans male	2
Trans femme	1
Trans woman	1
Genderqueer	1
Race and Ethnicity	
White	7
Latino/a/x	1
Black/African American	1
Asian/Asian American	1

### Procedure

**FIG. 1.**
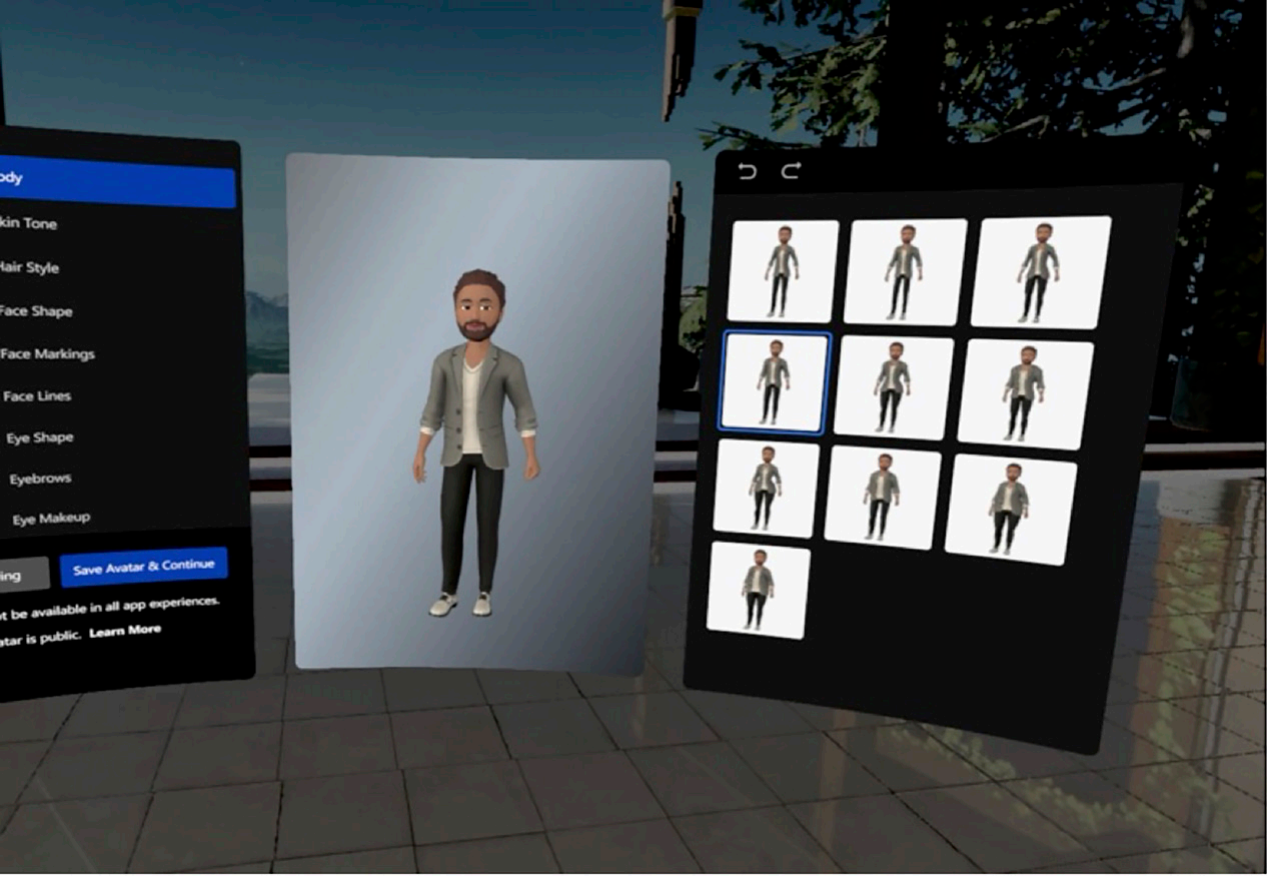
Avatar customization tools are not categorized by binary gender.

The groups, which were approved by the Human Subjects Board at Yale School of Medicine, consisted of five participants each and one clinical psychology postdoctoral fellow, and met once a week for eight weeks. The clinical psychology fellow was supervised and moderated by a licensed clinical psychologist with expertise in evidence-based care for TGE youth. A total of 16 group sessions were held between the two groups (eight per group) and group sessions lasted for 50–60 minutes, with a goal of sessions occurring once a week. If fewer than three participants were present for a session, the session would be postponed to the following week. Each participant’s Oculus Quest 2 headset was linked to a unique, deidentified account created and maintained by the first author using only assigned participant numbers. Each participant was encouraged to customize their avatar throughout the study, including their physical appearance and the pitch of the voice filters. Avatar customization was not categorized by gender and all participants had access to all body types, hair styles and lengths, facial hair, and make up options (Fig. 1). Similarly, voice filters were labeled with high or low pitch.

Given the need for confidentiality, a VR platform that appropriately maintained participant privacy was required. We used Foretell Reality, a social VR platform that is compliant with the Health Insurance Portability and Accountability Act and allows users to engage with others in an invite-only environment that does not store any personal identifiable information (Supplementary Video). The Foretell Reality platform also includes options for increased accessibility such as closed captioning, customizable text size, colorblindness optimization, and a single hand mode through which users with limited motor abilities can navigate the platform.^25^

[Supplementary Video, a promotional video showcasing the Foretell Reality platform, featuring the login process and avatar customization options].

The groups met in a virtual, curated space designed to be minimally distracting and maximally calming. Each individual user embodied their own custom designed avatar that was present in a circle with the other participants (five other patients and one group facilitator). The embodied avatar responded to the user’s movements and speech with six degrees of freedom. The user experienced the space with the assistance of the headset and hand controls as well as spatial audio to better sense where audio cues were originating.

During the groups, youth were encouraged to use first or affirmed names only to further maintain confidentiality. Within the Foretell Reality platform, user defined names and pronouns were displayed above the participants’ avatars. At the start of the first group for each cohort, we collaboratively established group rules including how the group would like to address confidentiality and offline interactions, if those arose (Table 2). The group facilitator opened the final guideline to each group, despite meeting in a VR space, as our participant sample comprised youth from a single clinic in the same state who may attend the same school. Therefore, the participants were given the option to discuss how they would like to handle this potential situation during the first group meeting.

**Table 2.** Group Guidelines Established by Group #1

1. Confidentiality: What is said here, stays here.
2. Please do not share any private information about other group members.
3. Respect: Respect pronouns, identities, and journeys.
4. Ask for permission before providing advice or feedback to another group member.
5. Step up, step back. If you know you are a talker, we absolutely want to hear from you, we just ask that you give folks who don’t talk as much a chance to step up too. Everyone deserves to be heard. Every voice matters in group.
6. One Rockstar, one mic. If someone is speaking, we want to let them have the floor and make space for each person to share to the level of their comfort.
7. What do we do if we see each other outside of group?

At each subsequent group, the guidelines were reviewed, and participants were offered the opportunity to add or change the rules as a group. During the eight-week groups, the participants were provided with opportunities to share about their lives and give and receive support from the group, as well as engage with different activities including drawing in three dimensions and playing catch with a virtual ball. Participants were also able to explore novel VR environments as a group, including an island/beach setting and a campfire setting where participants could roast marshmallows and take turns playing a virtual guitar. For the duration of each group, technical support was available to all participants.

At the conclusion of the last session, participants returned the headsets to the principal investigator and each headset was disinfected through the use of isopropyl alcohol wipes on the appropriate surfaces as described in a recent study by Roberts and colleagues.^26^ To further preserve confidentiality, each headset was factory reset between groups and new accounts were created for incoming participants.

### Risk management and safety procedures

Given the novelty of this intervention, it was important for the research team to consider risk factors and develop specific safety procedures. While supportive rather than therapeutic in nature, the groups provided participants with opportunities to discuss their lived experiences, which may have included significant struggles with mental health, gender dysphoria, and societal stigmatization. Each participant was provided with contact information for local, state, and national resources for crisis intervention. Further, the facilitator obtained emergency contact information from each participants’ caregivers, as well as the participant’s physical address. Each participant was also provided with a direct phone number to the facilitator. If any youth participant disconnected unexpectedly and did not respond to the facilitators’ attempts to re-establish contact, emergency services would be contacted. In addition, if any participants expressed significant psychological distress, including suicidal or homicidal ideation, emergency services would be notified. It is important to note that no mental health crises were reported during the sessions.

### Measures

Participants were given questionnaires prior to the first group meeting (baseline) and immediately following the final group meeting. Data were compiled using the Qualtrics software application and included a demographic questionnaire, the System Usability Scale (SUS), and a qualitative survey about participant experiences in the group (Table 3). The SUS is a well-validated, 10-item scale used to assess the usability of a product or service.^27^ For this study, we asked participants to respond to the SUS with both the Oculus Quest 2 headset and the Foretell Reality platform in mind. Feasibility was evaluated through attendance and retention rates.

**Table 3.** Qualitative Survey Questions

1. What parts or aspects of the group were your favorite?
2. What parts or aspects of the group did you not enjoy?
3. Did having an avatar change the way you felt about interacting with other participants? If so, how?
4. Please explain any impact (good, bad, neutral) having an avatar made on your overall experience in group?
5. If you could change anything about the group, what would you want to do differently?
6. How did you feel about the number of people in the group?
7. How do you feel about the content that we discussed in group?
8. What topics do you feel would have been more helpful to discuss in group?

### Data analysis

Participant responses were analyzed by a clinical psychology postdoctoral fellow and a clinical psychology predoctoral fellow to investigate the themes of acceptability and accessibility. Themes were reviewed by senior investigators. As participants responses were brief, we utilized a thematic analysis to examine the potential patterns or themes present in the responses. Using a grounded theory approach, analysis was conducted using three steps, open coding, axial coding, and selective coding.^28^ During open coding, two broad categories emerged related to the acceptability and accessibility of the intervention. In axial coding, subcategories were formulated around the hardware, the software, and the VR interactions with other TGE youth. During selective coding, the subcategories were further organized into themes. Further unprompted themes emerged from participant responses related to affirmation of gender identity and increased social connectedness. A theme was established if the majority of participants endorsed it (five or more out of a possible 10). Responses were compiled and we provided a count of how many participants responded within each theme. Participant responses to the SUS were scored and are reported in percentile ranks. Percentile ranks above 68 indicate above average perceived usability.^27^ Participant attendance was collected for each participant in each group to analyze attendance rates and retention was defined by attending five or more group sessions.

## Results

### Accessibility of the intervention

Within the participant responses, the theme of accessibility was present regarding the VR technology, both the Oculus Quest 2 headsets and the Foretell Reality platform. Most participants (*n* = 8) ranked the VR intervention above average for perceived usability, with SUS percentile ranks above 68. Some participants expressed concerns related to technical difficulties, with several noting that unstable internet connections resulted in disruptions in group (*n* = 5) and that the avatars made it difficult for them to read particular social cues (i.e., knowing when another participant was about to speak) (*n* = 4). Overall, most participants reported that they were able to learn to use the technology quickly (*n* = 9) and without significant technical assistance (*n* = 8).

### Acceptability of the intervention

All participants reported that the VR intervention is acceptable for the intended purpose of a social support group (*n* = 10). The participants noted that the VR intervention positively impacted their ability to connect with their peers. Specifically, half the participants noted that the VR software, including the avatar customization tools and voice modulations features, made it easier for them to interact with their peers (*n* = 5). One participant noted that the VR environment was “better than being on Zoom” and that they “preferred having an avatar to having a webcam on.”

Most participants reported that they would use the VR intervention again (*n* = 8) and half of the participants shared that the VR intervention was one of their favorite aspects of the groups (*n* = 5). Most notably, a majority of participants reported that they did not want a specific therapy group but rather endorsed a desire for a less structured social support group (*n* = 8); as stated by a participant, “less talking about mental health…I already have a therapist…I want friends.”

### Feasibility of the intervention

Participant attendance rates for each group are presented in Table 4 as percentages of attended sessions. Each group was scheduled for a total of eight sessions and were, overall, well attended. In Group 1, two sessions were postponed due to technical difficulties experienced by participants, including internet connectivity and account log in issues. After a consultation with the technical support team at Foretell, participants experiencing technical difficulties met with a member of the research team individually to provide customized support. We further created a document with a list of common technical difficulties and steps to resolve them. It is notable that Group 2 did not require any sessions to be postponed due to technical difficulties, as the technical support document was reviewed and provided to participants prior to the initial group session.

**Table 4.** Participation Rates

Participant	Sessions attended	Percent attended
Group 1		
YGP01	8	100
YGP02	8	100
YGP03	7	87.5
YGP04	6	75
YGP05	8	100
Group 2		
YGP06	8	100
YGP07	7	87.5
YGP08	8	100
YGP09	6	75
YGP10	8	100
Average attendance across both groups		92.5

In terms of retention, all participants engaged in five or more sessions. However, it is important to note that one participant in Group 1 (YGP04) did not attend the final two sessions as they reported a need for a more structured therapeutic environment and did not feel that the group was able to meet that need. This participant was provided with referrals for structured mental health therapy groups in their area and engaged in a debrief with a group facilitator. The participant reported a desire to complete the post group measures and was provided with that opportunity. In Group 2, one participant (YGP09) was not able to participate in two sessions due to a medical event but continued participation once they were able.

### Gender affirmation and social connection

Through the participants’ responses to open-ended questions, themes surrounding gender identity affirmation (gender joy) and social connection were observed. Nearly all participants expressed that meeting other TGE youth was a positive outcome from the group (*n* = 9), with one participant noting that her favorite part of group was “meeting other trans people, everyone was so nice!” and all participants reported a desire to meet each other in person at the end of the groups (*n* = 10). Participants also endorsed that their avatars and the voice filters encouraged them to speak more (*n* = 5), avoid being mis-gendered (*n* = 4), and engage more easily in a group setting (*n* = 5). A participant shared that “I talked more than I do in person” due to the voice filters while another participant expressed that his favorite part of group was “not being misgendered…not even once!”

## Conclusions

As resources for TGE youth and their caregivers in the United States continue to be limited and restricted, the need for safe spaces and attractive interventions that promote safety and affirmation for TGE youth is evident. Using VR interventions, we can respond to the current attack on gender affirming care and actively promote gender joy and community connection for TGE youth. In this pilot study, we learned that the participants found the VR intervention to be accessible, acceptable, and feasible. Further, we found that a majority of our participants found the VR intervention to be affirming of their identities and allowed them to feel more comfortable to connect to their peers. The participants, nearly all expressed the desire, however, for a change in the structure of the group with an emphasis on connecting at a peer-to-peer level, without the need for a structured, moderated support group. The study team determined it was essential to prioritize the needs of the participants by actively incorporating their feedback. In light of the rapidly evolving sociopolitical landscape, it was important for the research team to remain adaptable and responsive to ensure that the intervention was tailored to reflect the participants’ experiences.

Prior to the recruitment for the VR-based support group, there was a noticeable drop in participation in video-based virtual support groups offered through the Yale Pediatric Gender Program. This drop in participation was observed as the support group shifted from in-person meetings to virtual, video-based meetings due to the COVID-19 pandemic. In the virtual support group meetings, it was noted that youth who did participate would keep their cameras off and communicate mostly through the group chat feature. The changes in communication and engagement were contextualized with an understanding that, for some TGE youth, seeing themselves on camera and being perceived by others in video-based platforms may activate gender dysphoria. In turn, the activation of gender dysphoria may have made it more difficult or psychologically taxing for those youth to engage. In addition to the activation of gender dysphoria due to being perceived on camera, TGE youth may also have experienced increases in gender dysphoria related to their voices. Voice is an important aspect of gender identity and can impact the ways in which a person with gender dysphoria is perceived.^29^ TGE youth in video-based groups may have utilized the group chat feature to mitigate experiences of dysphoria related to their voice and to avoid the risk of being misgendered. Through VR, these youth were given the opportunity to embody an avatar and utilize voice filters to reduce the likelihood of activating gender dysphoria.

The central limitation of this pilot study is the low number of participants, which reduces generalizability. The TGE youth that were recruited for the pilot came from one central location and further groups will be conducted to both increase the sample size and to reach more TGE youth from diverse lived experiences and backgrounds. Detailed demographic information, such as ability status and regional location, was not gathered but may be helpful for future groups to ensure accessibility and understand the impact of VR interventions for individuals who live in varied sociopolitical contexts. Information related specifically to gender joy, wellbeing, and social connection was not directly measured and will be gathered in future groups, through both qualitative survey questions and validated questionnaires, including the Social Connectedness Scale Revised and the EPOCH Measure of Adolescent Well-Being.^30,31^ Continued groups will also seek to utilize a participatory research model in which the study team will more closely collaborate with TGE youth participants to uplift and center their voices and better meet their needs.

While the technology itself was enthusiastically accepted by the participants, this is not to say that it was without challenges. Examples noted by our participants included unstable internet connections and difficulty in reading social cues. Connection issues are to be expected in a peer-to-peer connection schema, particularly when users are connecting from various locations, networks, and environments. As the nation’s 5G networks and broadband access improves, we expect these challenges to improve. Considerations for moving forward would include a dedicated edge computing network, however, this is not feasible at this time. The comments regarding lack of ability to read social cues are more directly actionable and we expect to improve in the short term. Prior versions of the hardware used provided only three degrees of freedom, meaning that tracking only occurred for turning left/right, looking up/down, and tilting the view. Current hardware allows for six degrees of freedom, adding tracking for movement to the left/right, forwards/backwards, and upwards/downwards. While this helps greatly with immersion and embodiment, its improvements on nonverbal communication are minimal. Moving forward, headsets currently in development are focusing further on bringing nonverbal communication to the digital, virtual space. These advances include eye tracking (making virtual eye contact more natural and convincing), tracking of facial expressions, and more detailed tracking of the hands, down to each finger.

The strengths of this pilot study include the ability to provide the technology directly to the participants to use in their homes and direct communication between the research team and the development team of the VR app company to provide real-time feedback to support the groups. For example, the research team was able to deliver participant feedback from the first group to the app developers that allowed for avatar design elements not being categorized by gender; a feature that the participants in the second group noted as being affirming. In addition, these groups were offered at no cost to the youth and all VR equipment was provided for the duration of the study to promote accessibility. By utilizing a VR environment and customizable avatars, the participants in our study were able to embody their affirmed identities in a community with their peers. By allowing and encouraging participants to modify their avatars as they saw fit while also ensuring that participant pronouns were easily visible, we offered the opportunity for our participants to explore and experiment with affirming gender expression while decreasing the risk of being misgendered. Another strength of this study includes the qualitative feedback from participants which raised a unique and important consideration for the study team. Namely, a majority of participants expressed a desire to connect with their peers rather than a specific group therapy environment. This initial feedback from participants in the first group allowed the study team to offer a less-structured group environment for the second group, however, with continued and consistent feedback regarding this matter, a conscious decision was made to discontinue sessions designed as support groups with a health professional and enroll all future groups in a peer-to-peer, VR-driven model. Thus, future studies will seek to further prioritize social connection between participants, as well as continually integrate participant feedback in the study design and implementation.

Further research is needed to understand the ways in which VR technology can be utilized to support the social connectedness of TGE individuals across the lifespan, as well as the ways in which VR technology could be added to existing therapeutic interventions for TGE populations as the need for telehealth increases. Continued research should seek to investigate the impact of VR on both decreasing gender dysphoria and increasing gender euphoria. Finally, as TGE youth continue to experience the loss of critical social support systems, VR interventions that provide safe and accessible supports are crucial. VR support groups take place solely in a virtual space and may offer TGE youth in unsafe external environments the opportunity to connect with peers and experience affirmation of their identities, while maintaining confidentiality and appropriate moderation. A peer-based model of social support groups on alternative VR platforms may provide TGE youth with a valuable opportunity for community connection that extends beyond the scope of a mental health setting. Future research could explore the effectiveness and impact of well-moderated, peer-supported environments that are not restricted by invasive regulations or diagnostic criteria.

## Abbreviations Used

LGBTQ: Lesbian, Gay, Bisexual, Transgender, and Queer
SUS: System Usability Scale
TGE: Transgender and gender expansive
VR: Virtual Reality
